# A cross-sectional assessment of the relationship between sedative medication and anticholinergic medication use and the movement behaviour of older adults living in residential aged care

**DOI:** 10.7717/peerj.9605

**Published:** 2020-07-24

**Authors:** Gaynor Parfitt, Dannielle Post, Lisa Kalisch Ellett, Renly Lim, Alison Penington, Megan Corlis, Elizabeth Roughead

**Affiliations:** 1 Allied Health and Human Performance, Alliance for Research in Exercise, Nutrition and Activity (ARENA), University of South Australia, Adelaide, South Australia, Australia; 2 Quality Use of Medicines and Pharmacy Research Centre, Clinical and Health Sciences, University of South Australia, Adelaide, South Australia, Australia; 3 Helping Hand Organisation, Adelaide, South Australia, Australia

**Keywords:** Sedative load, Older adults, Movement behaviour, Physical activity, Residential aged care, Medication & movement behaviour

## Abstract

**Objectives:**

Medications with anticholinergic or sedative effects are frequently used by older people but can increase risk of falls and adverse events; however, less is known about their effect on movement behaviour. Here we examine the cross-sectional association between medication use and movement behaviour in older adults living in residential aged care.

**Materials and Methods:**

Twenty-eight older adults living in residential aged care in metropolitan Australia participated. Medication data were collected from participants’ medical charts and sedative load and anticholinergic burden were determined. Seven-day movement behaviour was objectively assessed by a wrist-worn triaxial accelerometer. Raw accelerations were converted to sleep, sedentary time, and time in light, moderate, and moderate-to-vigorous physical activity. To explore the relationship between medication and movement behaviour, Spearman’s Rho correlations were conducted, as the data were not normally distributed.

**Results:**

Analyses indicated that while anticholinergic burden was not associated with movement behaviour, sedative load was negatively correlated with a number of variables, accounting for 14% variance in moderate-to-vigorous physical activity (MVPA), and 17% in the bout length of MVPA (*p* < .02).

**Conclusion:**

The findings of this study showed a negative association between sedative load, due to medicines, and an individual’s movement behaviour. The impact of this could be a reduction in the ability of this population to maintain or improve their functional mobility, which may overshadow any benefits of the medicine in some circumstances.

## Introduction

Multiple medicine use is common in older people, particularly in the aged care sector where studies indicate older people are taking between nine and 11 medicines, on average (Elliott & Woodward, 2011; McLarin et al., 2016; Roughead, Gilbert & Woodward, 2008). Medicines with sedative and anticholinergic activity are commonly prescribed for residents in aged care and include medicines for pain, behavioural symptoms of dementia, depression, anxiety, urinary incontinence and insomnia. The impact of sedation is multifaceted. There is evidence that sedative medication adversely affects cognition for people living with dementia (Hukins, Macleod & Boland, 2019), and contributes to increased falls and increased risk of death (Lapeyre-Mestre, 2016). The effect is also cumulative; studies show that risk of falls increases with increasing numbers of sedative medicines (Pratt et al., 2014), that the risk of confusion increases with increasing numbers of sedative medicines (Kalisch Ellett et al., 2016), and the risk of confusion increases with increasing numbers of anticholinergic medicines (Jamsen et al., 2017; Kalisch Ellett et al., 2014).

An active lifestyle, incorporating light and moderate-to-vigorous intensity activity, is associated with lower mortality (Lapeyre-Mestre, 2016) and reduced risk of major mobility disability (MMD) (Mankowski et al., 2017), but unwanted side effects from medicines may impact on physical function, leading to reduced physical activity. Use of medicines with sedative or anticholinergic properties has been shown to be significantly associated with poorer functional outcomes when evaluated using traditional research measures including activities of daily living, grip strength and balance tests (Hukins, Macleod & Boland, 2019; Pratt et al., 2014). Accelerometer data, to evaluate the relationship between medicines and movement behaviour, have not been studied. Further, there is limited information on the movement behaviour of older adults living in residential aged care. A recent study on older adults living in residential care that used accelerometer data to measure behaviour indicated individuals spent only 1% of their time in moderate-to-vigorous intensity physical activity (MVPA), 14% in low and light activity and 85% sedentary (Parry et al., 2019). If time in MVPA is associated with medicine use, then this may have implications for medication management and the assessment of the risk-benefit profile of medicines in older people.

The aim of this cross-sectional study was to report medicine use and movement behaviour and examine the relationship between sedative and anticholinergic load from medicine use and movement behaviour in a population of older adults living in residential aged care. It was hypothesised that time in MVPA would be correlated with medicine use.

## Materials and Methods

Participants in this study were residents living in two wings of a residential aged care facility (one a secure memory unit, the other a supported living unit), in a residential aged care facility in metropolitan Australia. Participants provided voluntary informed written consent to participate in the collection of movement behaviour data and to share their demographic data and medication records with researchers as part of an Exercise Physiology in Aged Care program (Parfitt et al., 2020). In cases where residents were unable to provide consent, voluntary informed written consent was provided by an individual resident’s next of kin or authorised representative. The residential aged care organisation provided ethics approval for the exercise program. The university’s Human Research Ethics Committee granted ethical approval for evaluation researchers to access participant data collected by and held at the organisation (protocol number 0000035728).

Medication data were collected from participants’ medical charts, during July and August 2016, prior to the implementation of the exercise intervention of the Exercise Physiology in Aged Care program. Anticholinergic load was determined using an adaptation of the method of Durán, Azermai & Vander Stichele (2013). Medicines on the Duran list that are available in Australia were scored unchanged from the Duran list. Medicines with anticholinergic properties that are available in Australia, but that were not included on the Duran list, were identified and anticholinergic burden was assigned by three clinical pharmacists and a geriatrician, based on the mechanism of action and pharmacological properties of the medicine. Sedative load was determined by summing the total number of sedative medicines used by each participant. Sedative medicines were defined as medicines which are required by law to be dispensed with a label warning that they cause sedation, as outlined in Appendix K of the Australian Poisons Standard (Australian Government Department of Health, 2019).

Movement behaviour was objectively assessed using the GENEActiv (ActivInsights Ltd., UK), a triaxial accelerometer, worn on the wrist, that records raw data accelerations. The staff of the aged care facility were trained in the process of applying and removing GENEActiv devices for the residents, in cases where the resident was not able to do so for themselves. Staff and participating residents were aware of the importance of continual wear of the device, with a log sheet used to record the time of removal and re-application. Only residents with minimum data were included in the analysis. GENEActiv accelerometers demonstrate high reliability, with coefficient of variations of 1.8% for intrainstrument and 2.4% for interinstrument reliability (Esliger et al., 2011). Participants wore the GENEActiv on their dominant wrist, removing it only for showering, medical scans, or hospitalisation.

### Data processing and planned statistical analysis

GENEActivs were configured to collect free-living data at 100 Hz for a period of seven days. At least sixteen hours of data each day, for at least four days out of the seven, were required to achieve minimum data. Using purpose-designed software (COBRA Processing), raw accelerations were converted to sleep, sedentary time, and time in light, moderate, and MVPA, using Esliger et al. (2011) cut-points. While cut-points were applied in accordance with the protocol established by Esliger et al. (2011), physical activity bout lengths were set at two-minutes rather than ten-minutes, as the older adults in this population were unlikely to be performing MVPA in bouts of 10-minutes or more (Jefferis et al., 2019).

To examine the relationship between movement behaviour and medicine use, data were first checked for normality using Kolmogoro-Smirnov and Shapiro–Wilk statistics prior to conducting correlational analyses. For normally distributed data Pearson’s bivariate correlations were to be computed. If data were not normal, Spearman’s Rho correlations were computed. Significance level was set at *α* < 0.05. SPSS version 26 (IBM corp., Armonk, NY) was used for all analyses.

## Results

Twenty-eight residents (mean 85.7 years (SD 7.05), 68 to 97 years; 23 females) participated in the study. Demographic data indicated that 89.3% of the cohort had reduced cognition (Psychogeriatric Assessment Scales scores (PAS) (Jorm et al., 1995)). The median number of medicines used per participant was 10 (interquartile range (IQR) 8–13). The median anticholinergic burden was 2 (IQR 1–4) and the median sedative load was 2 (IQR 1–3). The most frequently used medicines with sedative properties were risperidone (11 participants) and oxazepam (7 participants). The most frequently used medicines with anticholinergic activity were furosemide (14 participants) and risperidone (11 participants). Please see Table 1 for demographic data.

**Table 1:** Participant demographics, medication data, and movement behaviour (*n* = 28).

Variable	Mean or *n* (%)	Standard deviation or IQR
Age (Years)	85.7	7.05
Gender (Female *n* (%))	23 (69.7)	
Psychogeriatric Assessment Scale score (>5)	25 (89.3)	
Anticholinergic burden (median, IQR)	2	1–4
Sedative load (median, IQR)	2	1–3
Time: Sleeping/in bed (min)	636 min	87.4
Sedentary (min)	725 min	85.4
Light intensity (min)	64 min 18 s	69
Moderate intensity (min)	5 min 38 s	8.1
Moderate-to-vigorous intensity (min)	5 min 42 s	8.2
MVPA Bout length (min)	3 min 1 s	4.9
Number of MVPA bouts	1.02	1.6

**Notes.**

Values presented are means, *n*, or % and SD or IQR. *minutes per day will not equate to 1,440 due to non-wear time.

On average, across a day spanning 1440 mins, participants spent 44% sleeping/in bed. Over ninety percent of the time out of bed was spent in sedentary behaviour, 8% in light intensity activity, and less than 1% (0.7%) in MVPA. With the exception of sleep, variables were not normally distributed, therefore non-parametric Spearman Rho were computed to examine the hypothesis. There were no significant correlations between anticholinergic burden and movement behaviour; however, there were between the movement behaviour variables and sedative load for time in MVPA (*r* =  − 0.37, *p* < 0.05, *R*^2^ = 0.14), length of MVPA bout (*r* =  − 0.41, *p* < 0.05, *R*^2^ = 0.17) and number of MVPA bouts (*r* =  − 0.38, *p* < 0.05, *R*^2^-0.14). A higher sedative load was associated with less time active, shorter bouts and less bouts. Please see Table 1 for means and standard deviations.

## Discussion

The aim of this cross-sectional study was to describe the movement behaviour of the population and assess the impact of medication on movement behaviour in a cohort of people living in residential aged care. Results indicate that participants were sedentary, spending more than 90% of their day-time sedentary, and less than 1% in MVPA. Further, while anticholinergic burden was not associated with any components of movement behaviour activity, sedative load was. For this population, sedative load was associated with 14% variance in MVPA, and 17% for the bout length of MVPA (*p* < .05). This study demonstrates that sedative load is not only associated with the number of bouts of MVPA, but also the length of time spent in bouts of MVPA, such that individuals with a higher sedative load tend to engage in less MVPA and shorter bouts of MVPA. Due to this, the functional health benefits associated with physical activity are less likely to be achieved in this population.

This is a small cohort and limited to cross–sectional analysis, however, it is likely that the findings are representative of older adults living in secure memory units in residential aged care facilities. As such, if our results are applicable more widely, there are significant implications from these findings with regards to medication management and the assessment of the risk-benefit profile of medicines in older people. The association between medicine use and reduction in physical activity observed in this study suggests that the risks of reduced activity may outweigh any benefits of the medicine in some circumstances. The cumulative association also suggests that from a clinical perspective both dose and duration of use of sedative medicines should be minimised to avoid harms from decreased physical activity (Song et al., 2015; Theou et al., 2017). Future longitudinal analyses will be required to confirm this association. Our recent research in the residential aged care environment identified a decline in functional ability for older adults over a 12-week period (Parfitt et al., 2020). Anything that prevents older adults being physically active, such as sedative load, could further contribute to the trajectory of decline in this population.

The data also raises the importance of objective assessment of movement behaviour that is appropriate to the population of interest. The participants wore an accelerometer on their wrist for 24 hours each day, over seven days, to obtain objective high-resolution data. Data indicate that the participants were extremely sedentary, with on average just over an hour registered in light intensity activity and less than 6 minutes in MVPA over the course of 24 hours. These averages are lower than those reported by Parry et al. (2019), with a similar cohort, and worrying, given data indicate that increased sedentary time and decreased physical activity time are associated with increased risk of MMD (Mankowski et al., 2017). To comply with the World Health Organisation’s global physical activity international guidelines (World Health Organisation (WHO), 2011), the minimum bout length for MVPA is typically set at ten-minutes for adults; however, only a small percentage of older adults achieve this (Jefferis et al., 2019). As such, we modified the ten-minute bout length recommended by Esliger et al. (2011) to two-minute bout lengths because our population were residents in aged care facilities. This approach is supported by recommendations in the recently published UK Chief Medical Officers’ Physical Activity Guidelines (UK CMO Guidelines Writing Group, 2019), which indicates that the accumulation of shorter bouts lengths are just as beneficial for health outcomes in the older adult age group. Alteration of the bout lengths therefore ensured that we did not omit to record any meaningful sustained bouts of physical activity. Even so, the average number of bouts recorded of moderate-to-vigorous intensity was small, an average of 1 (SD 1.6).

In addition to the small sample size and cross-sectional study design, the non-parametric analysis is limited in its ability to account for confounders such as age and gender. That stated, these potential confounders were not correlated with medication use and are unlikely to explain the association between medication and movement behaviour in this study.

Besides the potential impact of medication, a possible explanation for low levels of recorded MVPA and moderate intensity physical activity could be the actual intensity cut-points. Research with older adults indicates they require greater energy expenditure, when expressed relative to their resting metabolic rate (Kwan, Woo & Kwok, 2004), than younger adults when performing the same task (Whitcher & Papadopoulos, 2014). Accelerometry cut-points need to take this into account. Research needs to establish appropriate cut-points for the raw accelerations of GENEActiv to apply to older populations to provide appropriate classification of time in different physical activity intensities. A change in intensity cut–points is unlikely to change our results on the association between medications with sedative effect on movement behaviour, however, it would ensure a more appropriate representation of actual movement behaviour in older adults.

## Conclusion

The need to prevent inactivity and provide opportunities for physical activity for older adults is recommended (UK CMO Guidelines Writing Group, 2019). This study has demonstrated that medications with sedative effects given to older adults in residential aged care are associated with decreases in movement behaviour. Other studies show that they are also associated with an increased risk of MDD (Mankowski et al., 2017), cognitive decline, falls, or death (Lapeyre-Mestre, 2016). Medicines are a modifiable risk factor and our results are suggestive that cumulative sedative load should be considered during medication reviews with the specific aim of minimising sedative load to support physical activity. Future longitudinal analyses will be required to confirm if this is a causal relationship.

##  Supplemental Information

10.7717/peerj.9605/supp-1 Supplemental Information 1 Anticholinergic load and sedative load, and movement behaviour for older adults living in residential aged care Age, gender, & PAS scores, plus anticholinergic and sedative load scores, and movement behavior values for 28 participants. The participant IDs were removed.

## Additional Information and Declarations

### Data Availability

The following information was supplied regarding data availability: The raw data is available in the Supplemental File.
